# Development of the Spence Children’s Anxiety Scale - Short Version (SCAS-S)

**DOI:** 10.1007/s10862-017-9637-3

**Published:** 2017-11-21

**Authors:** Johan Ahlen, Sarah Vigerland, Ata Ghaderi

**Affiliations:** 1 Department of Psychology, Uppsala University, Box 1225, 751 42 Uppsala, Sweden; 2 Department of Clinical Neuroscience, Karolinska Institutet, Stockholm, Sweden

**Keywords:** Anxiety symptoms, Children, Spence Children’s Anxiety Scale, Abbreviated questionnaire

## Abstract

The literature provides several examples of anxiety symptoms questionnaires for children. However, these questionnaires generally contain many items, and might not be ideal for screening in large populations, or repeated testing in clinical settings. The Spence Children’s Anxiety Scale (SCAS) is an extensively used and evaluated 44-item questionnaire developed to assess anxiety symptoms in children, and provides a sound base for the development of an abbreviated anxiety symptoms questionnaire. Although methodological standards have been presented in how to develop abbreviated questionnaires, previous studies have often suffered from several limitations regarding validating procedures. Guided by these methodological standards, the current study aimed at developing an abbreviated version of the SCAS, while retaining the content, convergent, and divergent validity of the original scale. A school-based sample (*n* = 750) was used to reduce the number of items, and an independent school-based sample (*n* = 371) together with a clinical sample (*n* = 93), were used to validate the abbreviated scale. The abbreviated version of the SCAS contained 19 items, it showed a clear factor structure as evaluated in the independent sample, and it performed as good as the original questionnaire regarding classification accuracy, convergent, and divergent validity. In our view, the abbreviated version is a very good alternative to the original scale especially for younger children, in initial screening, or in order to reduce response burden.

## Introduction

Several scientifically well-evaluated self-report questionnaires of anxiety symptoms in children have been described in the literature. However, they generally have contained a large number of items, which hamper their usability in various settings. There are several situations in research, schools, and clinical settings where a short, valid, and reliable questionnaire would be very beneficial. This paper concerns the development of an abbreviated version of the Spence Children’s Anxiety Scale, a widely used, and evaluated self-report questionnaire for anxiety symptoms in children.

### Literature Review

Anxiety disorders are frequent in children. Although prevalence rates vary remarkably between studies (3–24%), anxiety disorders are probably more common than both mood disorders, and behavior disorders in children (Baumeister and Härter 2007; Cartwright-Hatton et al. 2006). Many children with anxiety disorders are severely impaired in their daily life, and suffer from adverse effects on school performance and interactions with peers (Essau et al. 2000; Simon and Bögels 2009). Anxiety disorders have also been found to predict anxiety disorders and other psychiatric disorders later in life (Bittner et al. 2007).

Children may suffer from anxiety disorders without being identified (Monga et al. 2000). Research has shown that only about 20–30% of children with anxiety disorders utilize mental health services (Bienvenu and Ginsburg 2007; Essau 2005). Reasons might be that children with anxiety problems do not show as manifest difficulties as children with behavioral problems, and that teachers and parents do not recognize these problems unless they are very severe and cause significant difficulties (Monga et al. 2000). How to better identify children in need of referral to mental health services is an important challenge for primary health care and school health (Sourander et al. 1999). Structured interviews, most often used in clinical settings, are time-consuming and not practical for screening (Spence 1998). In contrast, questionnaires might be used for screening in a cost-effective manner (e.g. in schools). Preliminary results have showed that such screening identifies children not identified using traditional teacher referral systems (Eklund et al. 2009).

Several questionnaires of anxiety symptoms in children have been described in the literature; Beck Anxiety Inventory for Youth (BAI-Y; Beck et al. 2001), the Fear Survey Schedule for Children-Revised (FSSC-R; Ollendick 1983), the first and the second edition of the Multidimensional Anxiety Scale for Children (MASC & MASC2; March et al. 1997; March 2013, respectively), the Revised Children’s Anxiety and Depression Scale (RCADS; Chorpita et al. 2000), the first and the second edition of Revised Children’s Manifest Anxiety Scale (RCMAS & RCMAS2; Reynolds and Richmond 1985; Reynolds and Richmond 2008, respectively), the Screen for Child Anxiety Related Emotional Disorders (SCARED; Birmaher et al. 1997), the Spence’s Children’s Anxiety Scale (SCAS; Spence 1998), and the State-Trait Anxiety Inventory for Children (STAI-C; Spielberger 1973).

### Length of Questionnaires

Questionnaires of anxiety symptoms have typically contained a large number of items; MASC contains 39 items, MASC 2 50 items, RCMAS 37 items, RCMAS2 49 items, SCARED 41 items, STAI-C 40 items, FSSC-R 80 items, and SCAS 44 items. The length of a questionnaire has shown to be important to its usability (Ebesutani et al. 2012). For example, the completion rate is lower for longer questionnaires, and items towards the end of questionnaires seem to be answered in a more careless manner (Galesic and Bosnjak 2009). Martinussen et al. (2013) also reported that people with low educational level to a lesser extent complete long questionnaires. There are several situations in research, schools, and clinical settings where a short (but nevertheless valid and reliable) screening questionnaire of anxiety symptoms would be very beneficial (Billieux et al. 2012; Li and Lopez 2007). For example, in the process of screening in large school populations, the brevity facilitates the inclusion in regular checkups for schoolchildren. Moreover, the use of brief questionnaires to assess secondary outcomes in clinical trials is a good way to reduce response burden for the child.

### Abbreviated Questionnaires

The development of abbreviated versions of full-length questionnaires has been characterized by a lack of rigorous psychometric validating procedures (Smith et al. 2000). Researchers in psychology nevertheless often consider themselves required (by practical reasons) to use short versions rather than full-length questionnaires due to studying complex models comprising many constructs of interest (Smith et al. 2012). However, it is quite possible to develop valid abbreviated measures, not necessary less valid than full-length measures if adhering to sound methodological standards (Smith et al. 2012). Smith et al. (2000) presents nine methodological important procedures for developing abbreviated forms of questionnaires for clinical assessment; (1) examine the balance between time/resources saved and loss of validity, (2) select items to retain, based on a content analysis (preserve as much content coverage as possible), (3) administer the abbreviated form to an independent sample, (4) examine reliability in the new independent sample, (5) examine overlap between abbreviated and full-length forms, (6) examine factor structure for the abbreviated form, (7) validate the abbreviated form, (8) examine classification ability, and (9) if developing a uniform measure out of a multidimensional measure, conduct a content analysis to examine the possible narrowing of the construct.

There have been earlier attempts to create short questionnaires for anxiety symptoms.

The BAI-Y is a 20-item inventory of anxiety symptoms, and it has several strengths beyond its brevity. The BAI-Y has been evaluated in a large American stratified sample and total scale scores have shown good internal consistency and high test-retest reliability (Steer et al. 2001). However, the BAI-Y suffers from some limitations. First, no subscales of the BAI-Y have been suggested or examined, which raises the question to what degree the inventory capture the content of the multidimensional construct of anxiety (Bose-Deakins and Floyd 2004). Further, the evidence for discriminant validity of the BAI-Y is rather weak, due to results showing that the total scale scores being highly associated (i.e. *r >* .70) to inventories of depression (e.g. BDI-Y [Steer et al. 2001; Thastum et al. 2009]). Lastly, regarding classification accuracy, the BAI-Y has been found to poorly discriminate between children with anxiety and depression (Thastum et al. 2009).

A short form of the MASC (MASC-10) has been derived from the total scale (March and Sullivan 1999). The MASC-10 was created by taking the four highest loadings on each of the four subscales of MASC (physical symptoms, harm avoidance, social anxiety, and separation anxiety), and then the ten highest loading items from these on a one-factor model were chosen. The MASC-10 has shown fair internal consistency (.67) and acceptable test-retest reliability in a clinical sample (Rynn et al. 2006), and it suffers from several limitations. First, in the development of the MASC-10, it is not clear whether the short form adequately preserves the content of the subscales. Second, the MASC-10 has not been administered to an independent sample, other than embedded in the original scale. This implies that the factor structure, and reliability of scores and validity of the questionnaire has not been adequately examined.

Muris et al. (2002a) reduced the 47-item RCADS (Chorpita et al. 2000) to 25 items (20 items of anxiety symptoms and 5 items of depressive symptoms). The shortened version of the RCADS was created by removing items with inconsistent factor loadings according to an exploratory factor analysis. The shortened version of RCADS showed good fit to data according to a 5-factor model (generalized anxiety disorder, separation anxiety disorder, social phobia, panic disorder, and major depressive disorder) (Muris et al. 2002a). However, the shortened version of the RCADS also suffers from limitations. First, it has not been evaluated in a clinical sample, which makes it unclear whether the reduction has affected its clinical usefulness or classification ability. Second, the abbreviated form has not been administered to an independent sample, which implies that this short questionnaire, in similar to the MASC-10, has not been adequately examined.

Recently, Ebesutani et al. (2012) also made an attempt to reduce the RCADS using a more sophisticated test-reduction approach, using the Schmid-Leiman bi-factor model, to better retain the hierarchical structure of the questionnaire. Their version of 25 items (15-items anxiety symptoms and 10 items of depressive symptoms) showed acceptable ability to discriminate between participants with an anxiety disorder and participants without an anxiety disorder in a clinical sample. Even though in large parts an impressive study, including sophisticated methods, large school-based and clinical samples with demographic information, this abbreviated form was not administered in an independent sample, and thus contains the same limitations as described above.

### The Spence Children’s Anxiety Scale (SCAS)

The SCAS is a widely used self-report questionnaire, translated into at least 22 languages (Essau et al. 2011). The SCAS was originally developed to assess symptoms of anxiety in the general population (Spence 1998). Two advantages with SCAS in comparison to other questionnaires are that it was developed specifically for children (that is, not a” junior-version” of an already existing adult questionnaire), and it screens for symptoms of specific anxiety disorders (i.e. separation anxiety, panic disorder, social phobia etc. [Spence 1998]).

The SCAS has been psychometrically evaluated in numerous countries around the world; Australia (Spence 1998), China (Zhao et al. 2012), Japan (Ishikawa et al. 2009), South Africa (Muris et al. 2002b), Iran (Essau et al. 2012), Greece (Mellon and Moutavelis 2007), Cyprus, Germany, United Kingdom, Italy, Sweden (Essau et al. 2011), the Netherlands (Muris et al. 2000), and the United States (Whiteside and Brown 2008). Based on the above-mentioned studies, internal consistency of the total scale scores has been shown to be excellent, with a median α of .92. The convergent validity of the SCAS has been supported in different studies (e.g. Essau et al. 2002; Spence 1998). Strong correlations have been found to other questionnaires of anxiety symptoms (SCARED *r* = .85, RCMAS *r* = .71) and a moderately strong correlation has been found to the Children’s Depression Inventory, CDI (*r* = .48).

The factor structure of the SCAS has been extensively evaluated. Most commonly the factor structure has been evaluated using confirmatory factor analysis, where a model containing six correlated factors corresponding to the six subscales of SCAS generally has been found to provide the best fit (Essau et al. 2011; Spence 1998; Zhao et al. 2012). Evidence for a second order solution, where six first order factors load upon a second order factor, has also been found, and has been suggested to explain the correlations between the first order factors (Spence 1998). Only two studies have evaluated the SCAS with exploratory factor analysis (EFA) (Muris et al. 2002b; Spence 1998). In the original evaluation by the author of the SCAS, an EFA showed support for a 6-factor model, and most items (32 out of 38) loaded on the purported group factor (Spence 1998). In contrast, Muris et al. (2002b) found support of a 4-factor model, where group factors where constituted by items from several different subscales.

Recently, a method termed bi-factor modeling has been rediscovered as an effective method for understanding the multidimensionality of a measure (Reise 2012) and thus serves as an appropriate method for modeling the factor structure of a hierarchical measure such as the SCAS. To the best of our knowledge, no study has evaluated the SCAS according to a bi-factor model. However, as mentioned above, a similar questionnaire (the RCADS) has been evaluated using an exploratory bi-factor analysis (Ebesutani et al. 2012). Strong support was found for a general factor, and some support was also found for additional group factors, mimicking the purported subscales reflecting different anxiety disorders (i.e. separation anxiety, panic disorder, and generalized anxiety). However, the social anxiety subscale was divided into two factors (one factor reflecting embarrassment, and the other concerns of achievements), while the subscale of obsessive-compulsive disorder was not supported in the analysis.

### The Current Study

The current study aimed at developing an abbreviated version of the SCAS primarily for screening, repeated testing, and other settings where a long questionnaire would not be very feasible. The present study adhered to contemporary strategies in creating abbreviated questionnaires, and included thorough psychometric validating procedures. To ensure a valid reduction of items both empirically and theoretically, we examined the original SCAS in a bi-factor exploratory factor analysis, and we carefully analyzed the content of items relevant to retain. According to the previous studies of the factor structure of the SCAS, and the bi-factor analysis of the RCADS, we hypothesized that the bi-factor model in the current study would show strong support for a general factor, and reasonable support for additional group factors reflecting the subscales of the SCAS. As seldom performed in other studies, we also administered the abbreviated version of the SCAS in a large independent school sample to examine reliability of the scores and factor structure. Lastly, we additionally examined convergent and divergent validity, and classification ability for the SCAS-S (as derived from the full-scale administration) and the original SCAS in a clinical, and a school-based sample.

## Method

### Participants

The first school-based sample (hereafter called School Sample 1) was recruited from schools in Stockholm, Sweden. In order to create a nationally representative sample, covering the variations in socioeconomic status between areas, we selected schools based on data on parents’ educational level (The Swedish National Agency for Education 2015). A total 41 schools were asked to participate. Seventeen schools agreed, and a total of 1163 children from these schools were asked to participate. Parental written consent was required for participation in the study. The parents of 777 children (67%) consented, while 93 parents (8%) refused to consent, and 293 parents (25%) did not respond to the invitation. A total of 750 children completed all items of the SCAS and were thus included in the analyses. Included children were 8–13 years of age (*M* = 9.6). There were 371 girls (49.5%) and 379 boys (50.5%).

To create the second school-based sample (hereafter called School Sample 2), we sent an invitation letter to 20 schools from urban and suburban areas of Stockholm. Parental written consent was again required for participation in the study. Five schools agreed, a total of 754 children were asked to participate, and the parents of 392 children (52%) consented. A total of 371 children completed all items of the SCAS-S and were thus included in the analyses. Included children were 8–13 years of age (*M* = 11.0). There were 195 girls (53%) and 176 boys (47%).

The clinical sample (*N* = 93) was recruited nationally in Sweden through media advertisement, mainly in Stockholm and adjacent municipals, as a part of a randomized controlled trial of internet-delivered CBT for children with anxiety disorders (Vigerland et al. 2016). Included children were 8–12 years of age, (*M* = 10.1) with a principal diagnosis of generalized anxiety disorder, panic disorder, separation anxiety, social phobia or specific phobia (except for blood-injury, or injection phobia) according to DSM-IV criteria. Participants were excluded from the study if the child had an autism spectrum disorder or an attention-deficit/hyperactivity disorder, was severely depressed or had another acute psychiatric disorder. All the diagnoses were established through structured clinical interviews. There were 51 girls (55%) and 42 boys (45%).

### Procedure

Children in the School Sample 1 completed the original SCAS (44 items), and children in the School Sample 2 completed the SCAS-S (19 items) at their school within regular school hours. Children were asked to sit individually, and in order to facilitate the children’s understanding, the first author (a clinical psychologist) or two master level psychology students were present in the classrooms to read the instructions and items aloud, and to answer any questions, while children answered the questionnaires. All questionnaires were coded to ensure confidentiality. The vast majority (90%) of the children in School Sample 1 participated in a longitudinal intervention study (Ahlen et al. 2017). Within this longitudinal study, there were 119 children who scored more than 1.5 standard deviations above the mean of the sample on any of the SCAS subscales at baseline. A total of 55 of these children (46%) agreed to participate in a structured interview, and they, together with 50 randomly chosen children without high anxiety scores at baseline, were interviewed using the Mini International Neuropsychiatric Interview for Children and Adolescents (MINI-KID; Sheehan et al. 1998). The 55 children that were interviewed, did not differ from the remaining children with high anxiety scores in regard to age, gender, or SCAS scores (*p* = .74, *p* = .64 *p* = .75, respectively). The 50 randomly chosen children did not differ from other children without high anxiety scores in regard to age, gender, or SCAS scores (*p* = .42, *p* = .90, *p* = .14, respectively).

All children and parents in the clinical sample completed child and parent versions of SCAS online. Parents additionally completed the Strength and Difficulties Questionnaire. One to four weeks later, the child and at least one parent underwent face-to-face assessment using the Anxiety Disorders Interview Schedule (child and parent version; ADIS-C/P; Silverman and Albano 1996) with a research assistant/psychologist. The research assistants were last-year students in the Swedish five-year clinical psychology program, with completed one-year training in cognitive behavior therapy. The other assessors were experienced clinical psychologists.

### Measures

The Spence Children’s Anxiety Scale (SCAS; Spence 1998) is a self-report measure of anxiety symptoms and consists of 44 items. Six items are ‘filler items’, which serve to reduce negative response bias. The remaining 38 items are divided into six subscales; separation anxiety disorder, social phobia, obsessive-compulsive disorder, panic attack and agoraphobia, physical injury fears, and generalized anxiety disorder. All items are rated on a four-point likert scale ranging from never (0) to always (3), regarding the frequency with which the child experiences each symptom. Internal consistency of scores was excellent in the current sample regarding total scores (*α* = .92), and acceptable regarding scores of separation anxiety disorder (*α* = .73), social phobia (*α* = .74), obsessive-compulsive disorder (*α* = .71), panic attack and agoraphobia (*α* = .76), and generalized anxiety disorder (*α* = .77). However, the scores of the physical injury fears subscale did not show adequate internal consistency (*α* = .58). In the current study, total score mean and standard deviation for the School Sample 1 (*M* = 26.3, *Sd* = 15.2) were comparable to means and standard deviations of the same age-group, reported from school-based samples in Australia (Spence 1998), Japan (Ishikawa et al. 2009), South Africa (i.e. a middle-high SES sample; Muris et al. 2002b). However, the mean of School Sample 1 was slightly higher than samples in China (Zhao et al. 2012) and the Netherlands (Muris et al. 2000), and considerably lower than a sample in Greece (Mellon and Moutavelis 2007) and a low SES sample in South Africa (Muris et al. 2002b). Further, total score mean and standard deviation for the Clinical Sample (*M* = 35.2, *Sd* = 13.4) were largely comparable to other clinical samples, with somewhat lower means than Australian samples (e.g. Lyneham and Rapee 2006; March et al. 2009), and slightly higher means than a Dutch sample (Nauta et al. 2003).

The Spence Children’s Anxiety Scale - Parent version (SCAS-P, Spence 1999), consists of 38 items, formulated to correspond to the child version. The six positive filler items have been removed from the parent scale. SCAS-P has shown high internal consistency of scale scores (Nauta et al. 2004). In the current study, the internal consistency of total scores was .88.

The Strength and difficulties questionnaire (SDQ; Goodman 1997) was developed as a short screening instrument to measure children’s mental health in large populations. The SDQ has shown acceptable internal consistency in a Swedish sample (Smedje et al. 1999). The SDQ has shown high correlation to Child Behavior Checklist, which is considered to be a valid measure (Goodman and Scott 1999). In the current study, internal consistency of the subscale scores was acceptable regarding Emotional problems (*α* = .71), but only fair regarding Peer problems (*α* = .63).

The Anxiety Disorder Interview Schedule Child and Parent version (ADIS C/P; (Silverman and Albano 1996) is a semi-structured interview conducted with the child and parent separately to assess diagnostic criteria according to DSM-IV. The severity of each diagnosis is assessed with the Clinician Severity Rating (CSR) on an 8-point scale (1–8). A score of 3 or lower is considered as subclinical symptoms whereas a score of 4 or higher means that the criteria for diagnosis are fulfilled with regard to severity. The ADIS C/P has shown good to excellent kappa coefficients and excellent test-retest reliability (Silverman et al. 2001). A strong association between specific disorders according to the ADIS C/P, and empirically derived factor scores of the corresponding construct supports the concurrent validity of the ADIS C/P (Wood et al. 2002). In the current study, inter-rater reliability was found to be good (κ = 0.65) for presence of anxiety disorders and excellent for CSR scores of severity (ICC = 0.77).

The Mini International Neuropsychiatric Interview for Children and Adolescents (MINI-KID; Sheehan et al. 1998) is a brief diagnostic interview for children and adolescents covering affective disorders, anxiety disorders, obsessive-compulsive disorder, oppositional defiant disorder, etc. The MINI-KID has shown to produce similar results as other diagnostic tools, and an overall good inter-rater reliability between raters (Sheehan et al. 2010). In the current study, inter-rater reliability was found to be good between raters (κ = .71).

### Data Analysis

#### Factor Structure of the Original SCAS

As a starting point in the decision of what items to retain in the abbreviated SCAS, we performed a bi-factor EFA in School Sample 1 (*N* = 750), using the Schmid-Leiman orthogonalization (Schmid and Leiman 1957) comprising one general factor, and six group-factors. In the bi-factor model each item loads on a general factor representing a latent construct (e.g. anxiety). In addition, each item is also free to load on a number of specified group factors (e.g. separation anxiety, social anxiety) representing additional common factors that explain the variance not accounted for by the general factor. Unlike the correlated factors model and the higher-order model, the group factors are uncorrelated to each other, and to the general factor (Reise et al. 2010). The Schmid-Leiman orthogonalization is one procedure to attain the bi-factor model. In short, the procedure involves (1) extracting factors and performing an oblique rotation, (2) extracting a second-order factor based on the correlations of the primary factors, and (3) performing a transformation to create uncorrelated general and group factors (Reise et al. 2010). In comparison to the correlated factors model and higher-order models, the Schmid-Leiman bi-factor model holds the advantage of calculating how variance is distributed to a general versus group factors (Reise 2012). Factor analyses based on Pearson correlations do not provide sound results when applied to ordinal data (Basto and Pereira 2012), consequently, we conducted the EFAs based on polychoric correlations. To measure the goodness of fit of the model, we reported two fit indices appropriate for ordinal data; the Goodness of Fit Index (GFI) and the Root mean square residuals (RMSR) (Jöreskog and Sörbom 1981). GFI values over .95 and RMSR values below .05 were interpreted as a good fit as recommended by Schermelleh-Engel et al. (2003). We used the FACTOR software (Lorenzo-Seva and Ferrando 2006) to perform all EFAs.

#### Item Reduction

An item was retained in the abbreviated version of SCAS if an item (1) loaded high (>.30) on the general factor of anxiety, and loaded high (>.30) on a group factor, and (2) was considered to be prototypic for the target construct (disorder), rather than only related to the target construct. Decisions regarding criteria 1 were taken directly from the bi-factor EFA of the original SCAS, while decisions regarding criteria 2 were taken based on judgments by the authors, guided by the DSM-5 manual (5th ed.; DSM–5; American Psychiatric Association 2013) and clinical expertise. We retained a group factor, only if a minimum of three items fulfilled the criteria above, as often recommended (e.g. Marsh et al. 1998).

#### Factor Structure of the SCAS-S

We, then performed a similar Schmid-Leiman bi-factor EFA of the SCAS-S in School Sample 2 (*N* = 371). To examine the extent of uni- vs. multidimensionality, we calculated the explained common variance. However, although a scale might show evidence of multidimensionality, it does not directly infer a reliable interpretation of subscales (Brouwer et al. 2013). Therefore, in order to further examine the interpretability of sum of scores (general and group factors), we calculated the coefficient omega hierarchical (*ω*_*h*_), where only the common variance of the factor of interest is thought to underlie the score, and other sources of common variances are treated as error variance. Calculating the omega hierarchical thus helps to clarify the feasibility of group factors in the model, as well as assess how item content mainly reflects a general factor of anxiety, a specific group factor, or both (Reise et al. 2010; Reise 2012).

#### Reliability of Scale Scores

Brunner et al. (2012) have emphasized the incongruity of using the Cronbach’s alpha coefficient when dealing with multidimensional and hierarchically structured constructs, and have instead proposed the omega coefficient, an estimate of the reliability of the scores when all sources of common variance are taken into account. Thus, in addition to providing Cronbach’s alphas, we also calculated the coefficient omega (*ω*) for the general factor and group factors.

#### Classification Accuracy

In the interviewed subgroup of the School Sample 1 we examined the ability of the SCAS total score, and the SCAS-S total score (as derived from the full-scale administration) to classify children with or without any anxiety disorder. Further, in the Clinical Sample, we examined the ability of the subscales of SCAS, and the subscales of SCAS-S (as derived from the full-scale administration) to discriminate between different anxiety disorders. In these analyses, we used AUC-values from ROC-curves. The AUC (Area Under curve) is a common global method to quantify diagnostic accuracy. The AUC-value range from .5 (no better than chance), to 1.00 (perfect diagnostic accuracy). A value of for example .75 means that a random participant from the diagnostic group 75% of times, will have a higher score than a random participant from the non-diagnostic group (Zweig and Campbell 1993). Guidelines suggest that an AUC under .70 represent poor, between .70–.80 represent acceptable, between .80–.90 represent good, and over .90 represent an excellent diagnostic accuracy (Forty et al. 2009).

#### Convergent and Divergent Validity

In the Clinical Sample, we also examined correlations to severity (clinician severity ratings and number of diagnoses), and to parents’ ratings of anxiety, emotional problems and peer problems in order to examine the convergent and divergent validity of the SCAS and the SCAS-S (as derived from the full-scale administration). We performed all ROC-analyses and correlation analyses in IBM SPSS statistics 22. In addition, we compared dependent correlation coefficients using the “psych” package (Revelle 2015) in the R software program (R Core Team 2015).

#### Age Effects

Lastly**,** we calculated reliability of scale scores, and examined factor structure of the SCAS-S in separate analyses for children aged 8–10 (*n* = 184), and children aged 11–13 (*n* = 187) in order to examine age effects in Schools Sample 2.

## Results

### Development of the SCAS-S

Table 1 presents factor loadings of the original SCAS examined in the School Sample 1, according to a Schmid-Leiman bi-factor EFA. The bi-factor model, comprising one general factor and six group factor, showed a very good fit to the data (GFI = .99, RMSR = 0.033). The results of the item reduction procedure (as defined in the method section) are described below.

**Table 1:** Schmid-Leiman bi-factor exploratory factor analysis of the full length SCAS (One general factor and six group factors) in School Sample 1 (*N* = 750)

Subscale	Item content	General	Group 1 (SEP)	Group 2 (SAD)	Group 3 (OCD)	Group 4 (PD)	Group 5 (SP)	Group 6 (GAD)
*separation anxiety disorder*								
	Fear of being alone	.46	.37					
	Fear of being away from parents	.54	.57					
	Worry over family members	.56						.32
	Fear of sleeping alone	.49						
	Trouble going to school	.64						
	Fear of staying away overnight	.45	.40					
*social phobia*								
	Fear of tests at school	.48		.43				
	Fear of public facilities	.41						
	Fear of embarrassment	.50		.39				
	Worry over school performance	.52		.43				
	Worry what other people think	.63						
	Fear of speaking	.42						
*obsessive-compulsory disorder*								
	Repeated checking behavior	.40						
	Get rid of bad/silly thoughts	.53						.36
	Magic thoughts rituals	.53						
	Compulsive behaviors	.40			.48			
	Disturbed by bad thoughts	.64						
	Magic behavior rituals	.53			.39			
*panic attacks and agoraphobia*								
	Sudden trouble breathing	.51				.46		
	Shaking for no reason	.50				.52		
	Fear of public transports	.45						
	Fear of crowding	.53					.31	
	Scared for no reason	.59				.31		
	Dizziness for no reason	.53				.43		
	Fast heartbeats for no reason	.54				.49		
	Worry over becoming scared	.65						
	Fear of small places	.44					.36	
*physical injury fears*								
	Fear of darkness	.43					.32	
	Fear of dogs							
	Fear of doctors	.46						
	Fear of heights	.42					.56	
	Fear of insects	.31					.32	
*generalized anxiety disorder*								
	Overall worry	.56						.33
	Funny feeling in stomach	.48		.38				
	Overall fear	.56						
	Heartbeats when agonizing	.50						
	Worry over myself	.70						.34
	Shaky when agonizing	.64						
Explained total variance		26.3%	3.2%	3.4%	2.8%	3.7%	3.4%	2.4%
Explained common variance		58.1%	7.0%	7.5%	6.3%	8.1%	7.6%	5.3%
Omega Hierarchical (*ω*_*h*_)		.87	.27	.26	.27	.30	.28	.18

Factor loadings below .30 are omitted from the table. Item content is a summary of the item, rather than the actual wording of the item

*SEP* Separation anxiety Disorders, *SAD* Social anxiety Disorder, *OCD* Obsessive-compulsive Disorder, *PD* Panic Disorder, *SP* Specific Phobias, *GAD* Generalized anxiety Disorder

#### General Factor

All items except one loaded high (>.30) upon the general factor. With the exception of item 18 (fear of dogs) with a loading of .20, factor loadings ranged between .31 and .70 (*Mdn* = .51). Item 18 was removed as a potential candidate of items to retain in the abbreviated version.

#### Group Factor 1 (SEParation Anxiety Disorders: SEP)

Of the original items assumed to assess separation anxiety, three out of six loaded high on both the general factor and the group factor labeled as SEP in our analysis (item 5; fear of being alone*,* item 8; fear of being away from parents*,* item 44; fear of staying away overnight*).* All of these items were considered to be prototypic to separation anxiety disorder, and therefore retained in the SCAS-S.

#### Group Factor 2 (Social Anxiety Disorder: SAD)

Of the original items assumed to assess social phobia, three out of six loaded high on both the general factor and the group factor labeled as SAD (item 6; fear of tests at school*,* item 9; fear of embarrassment*,* and item 10; worry over school performance)*.* These three items were considered to be prototypic to social anxiety disorder, and therefore retained in the SCAS-S. In addition, one item originally assumed to assess generalized anxiety disorder (item 3; funny feeling in stomach), loaded high on the group factor labeled SAD. However, this item was not considered to be prototypic to social phobia, and was therefore not retained in the SCAS-S.

#### Group Factor 3 (Obsessive-Compulsive Disorder: OCD)

Of the original items assumed to assess obsessive-compulsive disorder, only two out of six loaded high on both the general factor and the group factor labeled as OCD (item 40; compulsive behaviors*,* and item 42; magic behavior rituals). Because only two items fulfilled criteria for inclusion, the OCD factor was not included in the SCAS-S.

#### Group Factor 4 (Panic Disorder: PD)

Of the original items assumed to assess panic attacks and agoraphobia, five out of nine loaded high on both the general factor and the group factor labeled as PD (item 13; sudden trouble breathing, item 21; shaking for no reason, item 32; scared for no reason, item 34; dizziness for no reason, and item 36; fast heartbeats for no reason). All of these items were considered to be prototypic to Panic Disorder, and therefore retained in the SCAS-S.

#### Group Factor 5 (Specific Phobias: SP)

Of the original items assumed to assess physical injury fear, three out of six loaded high on both the general factor and the group factor labeled as SP (item 2; fear of darkness, item 25; fear of heights and item 33; fear of insects). These three items were considered to be prototypic to Specific Phobias, and therefore retained in the SCAS-S. In addition, two items assumed to assess panic attacks and agoraphobia (item 30; fear of crowding, and item 39; fear of small places), loaded high on the general factor, and the group factor labeled SP. The item 30 was not considered to be prototypic to Specific Phobia, and therefore not included in the SCAS-S. However, the item 39 was considered to be prototypic to Specific Phobia, and therefore retained in the SCAS-S.

#### Group Factor 6 (Generalized Anxiety Disorder: GAD)

Of the original items assumed to assess generalized anxiety disorder, only two out of six loaded high on both the general factor and the group factor labeled as GAD in our analysis (item 1; overall worry*,* and item 22; worry over myself)*.* In addition, one item assumed to assess obsessive-compulsive disorder (item 19; get rid of bad/silly thoughts), and one item assumed to assess separation anxiety disorder (item 12; worry over family members), loaded high on the general factor, and the group factor labeled as GAD. However, both these items were considered to be prototypic to Generalized Anxiety Disorder, and therefore retained in the SCAS-S.

### The SCAS-S Factor Structure

A total of 19 items were retained in the abbreviated version, SCAS-S. To test the factor structure of the SCAS-S we conducted a Schmid-Leiman bi-factor EFA in the School Sample 2, comprising one general factor, and five group factors (due to the exclusion of the OCD factor). The results of the factor analysis are presented in Table 2. To summarize, the results of the factor analysis very nicely fitted the suggested bi-factor structure comprising one general factor and five group factors. First, all items loaded high upon the general factor. Factor loadings ranged between .31 and .70 (*Mdn* = .57). Second, all items regarding four constructs (i.e. SEP, PD, SP, and GAD) loaded high on the corresponding group factor. Factor loadings ranged between .32 and .67 for SEP, between .35 and .58 for PD, between .31 and .43 for SP, and between .31 and .47 for GAD. Regarding the group factor of SAD factor loadings ranged between .26 and .66, meaning one of the item (fear of embarrassment) did not load high on the corresponding group factor, or any other factor. Further, this item had a slightly stronger loading on the GAD group factor, but was retained in the SCAS-S for the estimation of classification accuracy, convergent and divergent validity. This decision was made in order to keep the SAD-factor in the model, and not to narrowing the content of the SAD-factor. Regarding total variance explained the factor solution of the SCAS-S explained a total of 53.7%, compared to 45.3% in the factor solution of the original SCAS. Measures of fit showed a very good fit to data (GFI = 1.00, RMSR = 0.026).

**Table 2:** Schmid-Leiman bi-factor exploratory factor analysis of the abbreviated SCAS-S (One general factor and five group factors) in school Sample 2 (*N* = 371)

Subscale	Item content	General	Group 1 (SEP)	Group 2 (SAD)	Group 3 (PD)	Group 4 (SP)	Group 5 (GAD)
*SEP*							
	Fear of being alone	**.44**	**.32**	−.01	−.10	.28	.13
	Fear of being away from parents	**.58**	**.41**	.05	.13	.11	.09
	Fear of staying away overnight	**.46**	**.67**	.04	.05	.03	.03
*SAD*							
	Fear of tests at school	**.55**	.05	**.66**	.01	.01	−.02
	Fear of embarrassment	**.49**	−.09	.26	−.01	.05	.28
	Worry over school performance	**.61**	−.04	**.33**	.07	.04	.25
*PD*							
	Sudden trouble breathing	**.55**	.00	−.02	**.58**	−.06	.02
	Shaking for no reason	**.62**	.07	.10	**.50**	.04	−.06
	Scared for no reason	**.70**	.03	.00	**.35**	.18	.15
	Dizziness for no reason	**.60**	−.13	−.02	**.47**	.08	.10
	Fast heartbeats for no reason	**.59**	.10	.02	**.55**	−.02	−.03
*SP*							
	Fear of darkness	**.54**	.05	.02	.04	**.43**	.05
	Fear of heights	**.49**	.12	.04	.07	**.32**	.03
	Fear of insects	**.31**	.02	.12	−.03	**.40**	−.15
	Fear of small places	**.45**	.00	−.04	.11	**.31**	.07
*GAD*							
	Overall worry	**.62**	−.12	.22	.08	.11	**.31**
	Worry over family members	**.57**	.22	.03	.01	−.03	**.47**
	Get rid of bad/silly thoughts	**.59**	−.04	.08	.14	.07	**.33**
	Worry over myself	**.62**	.09	−.02	.10	.07	**.42**
Explained total variance		30.5%	4.5%	3.7%	6.9%	3.7%	4.4%
Explained common variance		56.8%	8.3%	6.9%	12.8%	6.9%	8.2%
Omega Hierarchical (*ω*_*h*_)		.83	.35	.28	.35	.27	.24
Omega (*ω*)		.93	.75	.71	.90	.68	.82
Cronbach’s Alpha (*α*)		.88	.63	.70	.82	.59	.75

Item content is a summary of the item, rather than the actual wording of the item. The actual scale, in its correct format could be viewed and downloaded at www.scaswebsite.com

*SEP* Separation anxiety Disorders, *SAD* Social anxiety Disorder, *PD* Panic Disorder, *SP* Specific Phobias, *GAD* Generalized anxiety Disorder

Bold font indicates loadings greater than .30

### The SCAS-S Reliability and Dimensionality

Table 2 also reports the coefficients related to reliability of scale scores, and dimensionality of the SCAS-S examined in the School Sample 2. The reliability according to the omega and alpha values was good to excellent regarding the total scale scores, and acceptable to good regarding all group factors, except the SP-factor, where coefficients were somewhat lower. The explained common variance of the general factor was 57%, meaning somewhat less than half of the common variance was explained by the group-factors, thus indicating some multidimensionality of the SCAS-S. This was very similar to the original SCAS, where the general factor explained 58% of the common variance. The omega hierarchical coefficient of the general factor was high (.83), meaning that 83% of the total sum scores reflected a common trait. However, the omega hierarchical coefficients of group factors (when controlling for the general factor) were low, ranging from .24 to .35, meaning that the variation in subscale sum scores to a larger degree reflected variation in the general trait (i.e. anxiety) than actual variation of the specific content of the subscales.

### Gender Differences in the SCAS-S

Table 3 presents means and standard deviations for the total-, and subscale-scores of the SCAS-S for the total sample and divided by gender in School Sample 2. In a series of Welch two sample t-tests, we compared mean scores of the total-, and the subscale-scores between genders. The statistical details of these analyses are presented in Table 3, which revealed that girls reported higher symptom scores regarding total anxiety, and on all subscales.

**Table 3:** Descriptive statistics for the SCAS-S, total sample and divided by gender in School Sample 2 (N = 371), and mean comparisons between genders according to a Welch Two Sample t-test

Scale	Scores	Total Sample		Girls		Boys		Girls vs. Boys		
		*M*	*Sd*	*M*	*Sd*	*M*	*Sd*	*t-value*	*df*	*p-value*
SCAS-S	Total anxiety scores	14.7	8.6	17.2	8.9	12.0	7.3	6.09	366.5	<.001
	Separation anxiety disorder	1.9	1.6	2.2	1.7	1.5	1.4	4.58	367.6	<.001
	Social anxiety disorder	2.8	1.9	3.3	2.0	2.3	1.6	5.48	363.1	<.001
	Panic disorder	2.2	2.6	2.6	2.9	1.7	2.3	3.25	363.8	.001
	Specific phobias	3.8	2.6	4.5	2.6	2.9	2.2	6.45	368.5	<.001
	Generalized anxiety disorder	4.1	2.5	4.5	2.5	3.6	2.3	3.43	368.9	<.001

*SCAS-S* Spence Children’s Anxiety Scale – Short Version

### Classification Accuracy

Results from the ROC-curve analyses are presented in Table 4. A first ROC-analysis, based on the subsample of the School Sample 1 showed that the total sum scores of the SCAS, and the total sum scores of SCAS-S both performed acceptably (almost well), in classifying children into a diagnosis or no diagnosis.

**Table 4:** Classification accuracy for the total scale ^a^, and corresponding subscales ^b^ in the SCAS/SCAS-S

ADIS-C/P diagnoses (n = yes/no)	SCAS	SCAS-S
	AUC [95% CI]	AUC [95% CI]
Any anxiety disorder (*n* = 21/84)^a^	.78 [.66, .88]	.78 [.66, .88]
Separation Anxiety Disorder (*n* = 48/45)^b^	.76 [.66, .85]	.77 [.67, .86]
Social Anxiety Disorder (*n* = 37/56) ^b^	.78 [.68, .87]	.76 [.67, .86]
Specific Phobia (*n* = 76/17) ^b^	.85 [.75, .95]	.80 [.71, .90]
Generalized Anxiety Disorder (*n* = 41/52) ^b^	.63 [.52, .75]	.61 [.50, .73]
Panic Disorder (*n* = 6/87) ^b^	.70 [.50, .90]	.61 [.32, .90]

*AUC* Area under curve, *ADIS-C/P* The Anxiety Disorder Interview Schedule Child and Parent version, *SCAS* Spence Children’s Anxiety Scale, *SCAS-S* Spence Children’s Anxiety Scale – Short Version

^a^Any anxiety disorder was examined in Schools Sample 1

^b^specific disorders were examined in the Clinical Sample

All children in the Clinical Sample were diagnosed with at least one of the following anxiety disorders; Separation Anxiety Disorder, Social Anxiety Disorder, Panic Disorder, Specific Phobia, or Generalized Anxiety Disorder. There was a large rate of comorbidity in the sample, 26 children met criteria for one anxiety disorder, 31 met criteria for two anxiety disorders, 25 met criteria for three anxiety disorders, 10 met criteria for four anxiety disorders, and one child met criteria for five anxiety disorders. ROC-analyses showed that the SEP-, and SAD subscale sum scores of the SCAS, and the SCAS-S both performed acceptably in correctly classifying children into these disorders. Further, the SP subscale sum scores of the SCAS, and the SCAS-S performed well, in correctly classifying children into a diagnosis of specific phobia. Lastly, the PD and GAD subscale sum scores of the SCAS, and the SCAS-S both performed poorly, in correctly classifying children into these disorders.

### Convergent and Divergent Validity

Table 5 presents correlations between the total sum scores of the SCAS, the SCAS-S, the severity measures, and the parents’ ratings examined in the Clinical Sample. When we examined the correlation coefficients of the SCAS and the SCAS-S in regard to the association to overall clinician severity ratings, number of disorders, the SCAS-P, and the two SDQ subscales, we found no significant differences between the SCAS and the SCAS-S. Moreover, the total sum scores of the SCAS and the SCAS-S both showed a moderate to strong correlation to the parent’s ratings of the child’s anxiety according to the SCAS-P, suggesting convergent validity. The association to the Emotional Problem subscale was small to moderate, and seemingly (but not significantly) smaller than the association to the SCAS-P, (*p* = .08 and *p* = .09 for the SCAS and the SCAS-S respectively). However, the total sum scores of the SCAS and the SCAS-S showed significantly smaller correlations to the Peer Problem subscale, compared to the correlation to the Emotional Problem subscale of the SDQ (*p* = .01 for both the SCAS and the SCAS-S), suggesting divergent validity.

**Table 5:** Convergent, and divergent validity of the SCAS and the SCAS-S in the Clinical Sample

	SCAS-S	CSR	ADIS Diagnoses	SCAS-P	SDQ-Emo	SDQ-Peer
SCAS	.95***	.45***	.41***	.53***	.38***	.10
SCAS-S	–	.46***	.41***	.49***	.34**	.08
CSR^a^		–	.94***	.60***	.54***	.30**
ADIS Diagnoses			–	.55***	.55***	.27*
SCAS-P				–	.54***	.33**
SDQ-Emo					–	.46**

^a^the sum of all CSRs for the following diagnoses; Separation Anxiety Disorder, Social Anxiety Disorder, Specific Phobia, Generalized Anxiety Disorder, Panic Disorder

*SCAS* Spence Children’s Anxiety Scale, *SCAS-S* Spence Children’s Anxiety Scale – Short Version, *CSR* Clinician Severity Ratings in ADIS, *ADIS Diagnoses* Number of ADIS diagnoses, *SCAS-P* Spence Children’s Anxiety Scale – Parent Version, *SDQ-Emo* SDQ-Emotional problem subscale, *SDQ-Peer* SDQ-Peer problems subscale

*P* < .05; ** *p* < .01; *** *p* < .001

### Age Effects

Reliability coefficients and detailed results of the factor analyses for the different age-groups in School Sample 2 are presented in Appendix Tables 6 and 7. Internal consistency of total scale scores was good in both younger and older children (*α* = .89, *α* = .88, respectively). Further, in the younger children the internal consistency of scale scores was acceptable for GAD, PD and SAD (*α* = .76, *α* = .78, *α* = .70, respectively), and fair for SEP and SP (*α* = .62, *α* = .65, respectively). In the older children, the internal consistency of scale scores was good for PD (*α* = .85), acceptable for GAD (*α* = .75), fair for SEP and SAD (*α* = .64, *α* = .69, respectively), but poor for SP (*α* = .54). When we conducted a Schmid-Leiman bi-factor EFA with one general and five group factors in children aged 8–10, the five-factor solution previously presented in Table 2 (called the original model) was not completely replicated. The PD, SP and SEP factors in large resembled the corresponding factors in the original model, but some items loading on the GAD factor in the original model instead loaded on the SAD factor and vice versa. Due to this inconsistency, we also conducted a Schmid-Leiman bi-factor EFA with only four group factors in the younger children (see Appendix Table 8). In this model, the GAD and SAD factors merged into a single factor, while the remaining factors were similar to the previous model. Lastly, we conducted a Schmid-Leiman bi-factor EFA with five group factors in children aged 11–13. The results showed that all group factors in large resembled the corresponding factors in the original model except for the SP factor, where one item instead loaded on the SEP factor, and two items did not load high on any of the group factors.

## Discussion

### Summary

The purpose of the current study was to develop an abbreviated version of the Spence Children Anxiety Scale, while retaining the hierarchical structure, and validity of the original scale. The developed short version comprised 19 items, covering all subscales except obsessive-compulsive disorder from the original scale. The SCAS-S showed a very similar factor structure compared to the original scale, and performed as good as the original scale in all aspects of validity of the questionnaire and reliability of scale scores examined in the current study. We did not discover any general loss of validity, when comparing the abbreviated version to the original scale. Thus, examining the balance between loss of validity and time savings for the SCAS-S, with a reduction of 25 item, probably a time-saving of 10–20 min depending on child’s age, this clearly speaks for the SCAS-S in several settings.

When examining the dimensionality of the SCAS-S, we found evidence of multidimensionality. However, the omega hierarchical coefficients for the subscales scores ranged between .24 to .35, which undoubtedly indicate a restriction in the interpretation of the subscale scores as a direct measure of the specific disorder. Although reflecting the specific content to some degree, our analyses showed that all subscales to a larger degree reflected the general factor. Consequently, the implication is that the scores of the total scale could adequately be interpreted as a measure of anxiety, whereas the subscales only very cautiously could be interpreted as measures of the specific disorders. We recommend users of the SCAS-S to mainly use the total scale scores when screening for anxiety, and to use the scores of the subscales mainly as pointers in the process of additional clinical assessment. Further, we administered the SCAS and the SCAS-S by reading the items aloud in order to increase children’s understanding. Consequently, we recommend that clinicians and researchers follow the same administration strategy to not compromise the validity of the questionnaire.

The only item not loading high on both the general factor and the assumed group factor in the SCAS-S was one of the items of the SAD-factor (i.e. fear of embarrassment). Instead it loaded slightly higher on the GAD-factor. A possible explanation could be due to a narrowing of the construct in the item reduction procedure. More specifically, two items very prototypic to social anxiety disorder was not retained in the SCAS-S (e.g. fear of speaking, worry about what other people think). Consequently, the SAD-factor in the SCAS-S might only measure one part of the construct of social anxiety disorder, namely worry regarding school performance. This might have pushed the fear of embarrassment item towards the GAD-factor which contained several forms of worries.

As shown in Table 3, girls reported higher symptoms of anxiety compared to boys. Similar results were found by the author of the SCAS (Spence 1998), and have also been found in evaluations of the SCAS in other countries (e.g. Greece: Mellon and Moutavelis 2007; Japan: Ishikawa et al. 2009; South Africa: Muris et al. 2002b). As recommended by Spence (1998), we also suggest that scores of the SCAS-S are interpreted for girls and boys separately in clinical practice. The age-group-specific factor analyses showed some inconsistencies between the age groups. First, the SP factor was cohesive only in younger children. This might be explained by the fact that fears of darkness, animals etc. are more common in younger ages, and perhaps these items therefore to a larger extent cluster into a common factor. Moreover, the remaining factors (GAD, SAD, SEP, PD) were more cohesive in the older age group, which might be an effect of a more in depth understanding of the item content in older children.

Reliability coefficients appeared to be larger for the GAD and PD factors, which might to some degree be explained by the larger number of items in these subscales. Further, the seemingly lower reliability coefficient of the SP factor is somewhat expected since the grouping of different specific fears into a single factor is not as logical as the grouping of symptoms related to other anxiety disorders (i.e. to receive a diagnosis of Specific Phobia, it is sufficient to only have one specific fear).

The SCAS-S (and the SCAS) showed only a moderate ability to accurately classify children with or without an anxiety disorder. However, similar results have been found for other scales like the SCARED and the RCADS (e.g. Canals et al. 2012; Ebesutani et al. 2012), and somewhat lower AUC-values have been found regarding the MASC (Skarphedinsson et al. 2015; van Gastel and Ferdinand 2008). In the current study, AUC-values varied from poor to good regarding discriminating between specific disorders, and similar results were found in a study of the MASC (van Gastel and Ferdinand 2008). Slightly higher AUC-values were found for the SCARED regarding specific disorders (Canals et al. 2012). However, the latter study did not involve a clinical sample, which probably meant lower rates of comorbidity that might have affected these values. To clarify, a high degree of comorbidity is associated to higher total anxiety scores (see Table 5). Following the results of the bi-factor analysis in the current study, a large proportion of item-variances is accounted for by the general factor. Hence, as the variation of subscale-scores to a large extent is dependent on broad anxiety (i.e. the general factor), individuals with comorbidity will display higher scores even for disorder-specific subscales for which they don’t have a diagnosis, which might make it more difficult to discriminate between specific disorders. Furthermore, diagnoses in the study by Canals et al. (2012) were based solely on interviewing the child, and these AUC-values might therefore be somewhat larger due to shared method variance. To conclude, the SCAS-S seems to perform as well as other self-report measures of anxiety regarding classification accuracy. To further evaluate the classification accuracy of the SCAS-S and similar measures, studies should include diagnostic interviews based on separate child-, and parent-reports, and be executed in both clinical and non-clinical samples in order to control for shared method variance, and to examine the classification accuracy (and possible differences in accuracy) for different samples. Worth mentioning is that the accuracy differed between disorders with an acceptable ability to correctly classify disorders which typically have their onset in childhood (i.e. SEP, SAD, SP; see Kessler et al. 2005), but reduced ability to correctly classify disorders which typically have later onset (i.e. GAD and PD).

A strength in the examination of convergent and divergent validity is that the SCAS-S (child-ratings) were compared to parent measures, which reduces the bias of shared method variance. The correlations between the SCAS-S and the parent measures support convergent and divergent validity of the SCAS-S. Specifically, the strongest correlations were found between SCAS-S and SCAS-P, as one should expect, when measuring the same construct. Second, a less strong correlation (however not significantly lower) was found between SCAS-S and the SDQ-Emotional problem, a related construct containing questions of both anxious and depressive symptoms in the child. Third, a non-significant, and significantly smaller correlation were found between SCAS-S and the Peer-problem, a measure containing question regarding the child’s lack of friends and exposure to being bullied etc. However, in the current study, convergent and divergent validity was only examined in the Clinical Sample, and future studies of the SCAS-S should examine validity also in a normal sample.

In contrast to previous EFAs of the full length SCAS, many items (40%) did not load high on any group factor after controlling for variance accounted for by the general factor. Further, of the items that loaded on group-factors, five of them loaded on other subscales than purported (see Table 1). However, these items have also been found to load on different subscales than supposed, or cross-load in previous studies (i.e. two of them in Muris et al. 2002b, *worry over family members* and *get rid of bad/silly thoughts,* and two of them in Spence 1998, *fear of crowding* and *fear of small places*). Consequently, these items are as a suggestion not prototypic, rather related to the target construct, and the high loadings reported in previous CFAs might be due to the solid higher order factor (i.e. broad anxiety), and flawed constraints of the models.

### Limitations

Although the validation of the SCAS-S in part was performed in the independent sample, and administered in the actual short form, classification accuracy as well as convergent and divergent validity of the SCAS-S were examined using scores derived from the administration of the original SCAS. Smith et al. (2000) have emphasized the importance of examining the convergent and divergent validity of the actual short form, which only occasionally has been performed in research. It’s logical to find an overestimation of the similarity between the SCAS-S and the SCAS under this procedure, because the scores of the items in the abbreviated scale per definition are exactly the same as the scores of the same items in the full version. In order to more adequately examine the convergent and divergent validity of the SCAS-S, future research on the SCAS-S should therefore administer the actual short scale. Further, we excluded the subscale of OCD in the abbreviated version, due to not containing enough item with loadings above .30. This decision undoubtedly meant a narrowing of the construct, and is a significant weakness of the current study regarding the aim of preserving the content of the original SCAS. However, previous attempts to reduce dimensional anxiety measures for children have had similar problems with the evidence of an OCD factor. For example, in the study by Ebesutani and colleagues, no OCD-item loaded higher than .30 in their school-based sample, and in the study by Muris et al. (2002a), the OCD-factor was excluded for the same reasons as in our study (i.e. the factor only contained two items). Moreover, as reported by Mataix-Cols et al. (2007), OCD-experts typically consider intrusive thoughts and repetitive behaviors, rather than anxiety being the primary features of OCD, and a majority of OCD-experts agreed the transfer of OCD from the chapter of anxiety disorders to a separate chapter in the Diagnostic and Statistical Manual of Mental Disorders (5th ed.; DSM–5; American Psychiatric Association 2013). In the screening for OCD, we therefore recommend researchers and clinicians to use other brief screening-instruments specifically developed to screen for OCD, for example the well-established Obsessive–Compulsive Inventory-Short Version (OCI-R; Foa et al. 2002). Also, a limitation is that the subscale of social phobia in the SCAS-S appeared to reflect a narrower construct than in the full length SCAS. Moreover, a possible limitation of the study is that we used different samples to examine different aspects of validity. For example, the convergent and discriminant validity was only evaluated in the clinical sample, and the results are not simply generalizable to non-clinical samples. Lastly, we were unable to determine the test-retest reliability from the current data. In order to evaluate the reliability of repeated testing regarding the SCAS-S scores, we encourage future studies to distribute the SCAS-S at several occasions. With these limitations in mind, we believe the abbreviated version SCAS-S, is a good alternative to the original scale especially when completed by younger children, in initial screening, or in order to reduce response burden.

## Appendix 1

**Table 6:** Schmid-Leiman bi-factor exploratory factor analysis of the SCAS-S (One general factor and five group factors) in School Sample 2, age 11–13 (n = 187)

Subscale	Item content	General	Group 1 (SEP)	Group 2 (SAD)	Group 3 (PD)	Group 4 (SP)	Group 5 (GAD)
SEP							
	Fear of being alone	0.39	0.37			.33	
	Fear of being away from parents	0.54	0.36				
	Fear of staying away overnight	0.49	0.87				
SAD							
	Fear of tests at school	0.49		0.87			
	Fear of embarrassment	0.47					
	Worry over school performance	0.57		0.3			
PD							
	Sudden trouble breathing	0.54			0.70		
	Shaking for no reason	0.57			0.62		
	Scared for no reason	0.63			0.41		
	Dizziness for no reason	0.52			0.54		
	Fast heartbeats for no reason	0.56			0.59		
SP							
	Fear of darkness	0.45				0.49	
	Fear of heights	0.41	0.31				
	Fear of insects	0.34					
	Fear of small places	0.34					.
GAD							
	Overall worry	0.61					0.36
	Worry over family members	0.54					0.56
	Get rid of bad/silly thoughts	0.52					0.32
	Worry over myself	0.62					0.40
	Omega Hierarchical (*ω*_*h*_)	.79	.46	.32	.46	.23	.28
	Cronbach’s Alpha (*α*)	.88	.64	.69	.85	.54	.76

Factor loadings below .30 are omitted from the table. Item content is a summary of the item, rather than the actual wording of the item

*SEP* Separation anxiety Disorders, *SAD* Social anxiety Disorder, *PD* Panic Disorder, *SP* Specific Phobias, *GAD* Generalized anxiety Disorder

## Appendix 2

**Table 7:** Schmid-Leiman bi-factor exploratory factor analysis of the SCAS-S (One general factor and five group factors) in School Sample 2, age 8–10 (*n* = 184)

Subscale	Item content	General	Group 1 (SEP)	Group 2 (SAD)	Group 3 (PD)	Group 4 (SP)	Group 5 (GAD)
SEP							
	Fear of being alone	0.46					
	Fear of being away from parents	0.64	0.68				
	Fear of staying away overnight	0.47	0.34				
SAD							
	Fear of tests at school	0.54		0.30			
	Fear of embarrassment	0.44		0.48			
	Worry over school performance	0.64					
PD							
	Sudden trouble breathing	0.56			0.33		
	Shaking for no reason	0.63			0.40		
	Scared for no reason	0.71					
	Dizziness for no reason	0.65			0.32		
	Fast heartbeats for no reason	0.64			0.51		
SP							
	Fear of darkness	0.57				0.54	
	Fear of heights	0.49				0.38	
	Fear of insects					0.56	
	Fear of small places	0.49				0.30	.
GAD							
	Overall worry	0.56		.50			
	Worry over family members	0.64					0.46
	Get rid of bad/silly thoughts	0.62		.38			
	Worry over myself	0.63					0.40
	Omega Hierarchical (*ω*_*h*_)	.86	.27	.18	.22	.41	.11
	Cronbach’s Alpha (*α*)	.89	.62	.70	.78	.65	.75

Factor loadings below .30 are omitted from the table. Item content is a summary of the item, rather than the actual wording of the item

*SEP* Separation anxiety Disorders, *SAD* Social anxiety Disorder, *PD* Panic Disorder, *SP* Specific Phobias, *GAD* Generalized anxiety Disorder

## Appendix 3

**Table 8:** Schmid-Leiman bi-factor exploratory factor analysis of the SCAS-S (One general factor and four group factors). School Sample 2, age 8–10 (n = 184)

Subscale	Item content	General	Group 1 (SEP)	Group 2 (GAD/SAD)	Group 3 (PD)	Group 4 (SP)
SEP						
	Fear of being alone	0.46				
	Fear of being away from parents	0.62	0.37			
	Fear of staying away overnight	0.48	0.43			
SAD						
	Fear of tests at school	0.54		0.33		
	Fear of embarrassment	0.43		0.53		
	Worry over school performance	0.63		0.30		
PD						
	Sudden trouble breathing	0.56			0.33	
	Shaking for no reason	0.64			0.36	
	Scared for no reason	0.71				
	Dizziness for no reason	0.66			0.32	
	Fast heartbeats for no reason	0.65			0.50	
SP						
	Fear of darkness	0.56				0.46
	Fear of heights	0.48				0.42
	Fear of insects					0.61
	Fear of small places	0.48				0.32
GAD						
	Overall worry	0.55		0.43		
	Worry over family members	0.64				
	Get rid of bad/silly thoughts	0.62		0.41		
	Worry over myself	0.62		0.31		

Factor loadings below .30 are omitted from the table. Item content is a summary of the item, rather than the actual wording of the item

*SEP* Separation anxiety Disorders, *SAD* Social anxiety Disorder, *PD* Panic Disorder, *SP* Specific Phobias, *GAD* Generalized anxiety Disorder
